# The Effect of Alcohol Consumption on Progressive Supranuclear Palsy: A Cross‐Sectional Study

**DOI:** 10.1111/cns.70146

**Published:** 2024-12-23

**Authors:** Min Tian, Bohan Zhang, Heyin Liu, Yinlian Han, Mu Yang, Ruonan Duan, Dandan Guo, Chengyuan Song, Jun Ma, Yiming Liu

**Affiliations:** ^1^ Department of Neurology Qilu Hospital of Shandong University Jinan China

**Keywords:** alcohol consumption, cognitive function, motor symptoms, progressive supranuclear palsy, visuospatial and executive abilities

## Abstract

**Aims:**

To investigate the effect of alcohol consumption on the clinical symptoms in a cohort of Progressive supranuclear palsy (PSP) patients.

**Methods:**

We conducted a cross‐sectional study focusing on possible and probable PSP patients in Qilu Hospital of Shandong University. Diagnoses and clinical phenotypes were confirmed using the 2017 Movement Disorder Society criteria and the Multiple Allocations eXtinction (MAX) rules. Data on drinking habits and demographics were collected via face‐to‐face interviews and medical records reviews. Clinical scales assessed motor and nonmotor symptoms. Alcohol consumption was categorized into light, moderate, and heavy status. Using multivariate linear regression and adjusting for confounding factors, we analyzed the relationship between alcohol consumption and clinical symptoms.

**Results:**

The study comprised 128 participants (59.4% male and 45.31% drinkers). Alcohol consumption has been associated with severe PSP clinical symptoms, particularly among male patients. Compared with nondrinkers, consumers of alcohol exhibit significantly more severe motor symptoms and cognitive impairments, particularly in the domains of visuospatial and executive abilities, memory, and language. Moreover, when categorizing individuals based on their intake of alcohol weekly, those with heavy consumption show significantly higher PSP Rating Scale (PSPRS) and the Unified Parkinson's Disease Rating Scale (UPDRS) scores, as well as significantly lower Montreal Cognitive Assessment Scale (MoCA) and Mini Mental State Examination (MMSE) scores compared to nonconsumers.

**Conclusion:**

Our findings indicate an association between heavy alcohol consumption and more pronounced symptoms of PSP, especially cognitive function. It raises the possibility that alcohol intake may play a role in modulating the clinical course of PSP.

## Graphical abstract

An evaluative analysis conducted at our center on PSP patients has uncovered a significant association between alcohol consumption and more pronounced clinical symptoms. Specifically, heavy alcohol consumption exacerbates motor symptoms and accelerate cognitive decline, particularly in visuospatial and executive abilities, memory, and language functions. This correlation is particularly pronounced among male patients.

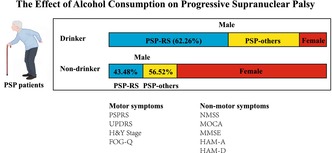

## 1 Introduction

Progressive supranuclear palsy (PSP) is a disabling parkinsonian disorder related to specific four‐repeat (4R) Tau neuropathology [1]. The core manifestation is characterized by vertical supranuclear gaze palsy, fall‐prone postural instability, bulbar symptoms, and cognitive dysfunction [1]. The etiology of PSP is complex, and it is hypothesized that genetic background and environmental factors are likely to underpin disease susceptibility [2, 3]. Identifying risk factors for PSP may contribute to a better understanding of its pathophysiology and to targeted prevention and treatment efforts.

The lack of definitive epidemiological data on PSP in China is notable, yet studies indicate that the prevalence of executive impairment in Chinese patients with PSP was frequent (76.9%), and it was associated with global cognitive dysfunction and disease disability [4]. Previous studies have concluded that heavy drinking and drinking more hard alcohol are linked to more rapid cognitive decline in people with Alzheimer's disease (AD) and moderate drinking reduces the risk [5, 6, 7, 8]. However, recent evidence suggests that even low levels of alcohol consumption may be associated with a reduction in brain volume and even an earlier age of onset of AD [9, 10, 11]. Chronic exposure to alcohol causes the phosphorylation of neuronal Tau in the hippocampus and impairs memory [5]. To our knowledge, there have been no dedicated studies specifically focused on the correlation between alcohol consumption and the clinical symptoms of PSP. Although a study of France has touched upon the broader topic of risk factors for PSP including drinking, they found no significant association between PSP and alcohol drinking [12]. The individual case report mentioned a PSP patient with alcohol‐related dementia [13]. Our work aimed to contribute to this area by examining the potential relationship between alcohol consumption and PSP symptoms in greater depth.

Here, we designed a study to investigate the effect of alcohol consumption (from light to heavy) on motor and nonmotor symptoms. We try to found the association between alcohol consumption and clinical symptoms of Chinese patients with PSP.

## 2 Methods

### 2.1 Study Population

**FIGURE 1:**
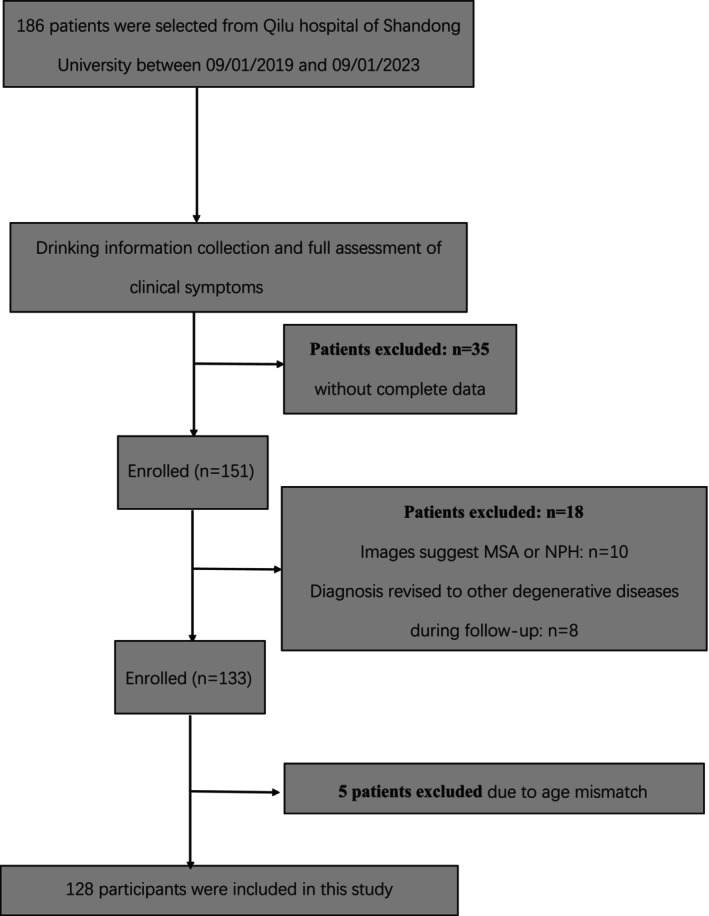
Flowchart of patient selection.

For this cross‐sectional study, participants were recruited at the Department of Neurology, Qilu Hospital of Shandong University between 09/01/2019 and 09/01/2023. All patients were diagnosed with “possible PSP” or “probable PSP” according to the Movement Disorder Society criteria for PSP and Multiple Allocations eXtinction (MAX) rules [1, 14], either at initial consultation or at follow‐up by experienced movement disorder specialists. We excluded subjects who had neurological diseases (e.g., Alzheimer's disease, other types of atypical Parkinsonism), serious systemic disorders (e.g., cancer), or psychiatric diseases (e.g., schizophrenia). 186 PSP patients were recruited. Thirty‐five patients failed to cooperate to complete the full assessment. MRI revealed significant cerebellar atrophy or ventricular hypertrophy in 10 patients, suggesting that those patients were Multiple System Atrophy or Normal Pressure Hydrocephalusge. Five patients were excluded due to age mismatch. And, in the course of the follow‐up process, the diagnoses of eight patients were revised. Ultimately, 128 patients with PSP were enrolled and analyzed (Figure 1).

The study was approved by the Institutional Ethics Committee (KYLL‐202008‐122‐3). Written informed consent was obtained from each participant or their legal representative.

### 2.2 Assessment

Information on drinking habits (an estimate of the quality of alcohol consumed daily and exposure duration) was collected by means of a face‐to‐face interview at recruitment. To ensure information accuracy, we also consulted the patients' family members and caregivers. We assessed alcohol consumption frequency and alcohol intake volume according to beverage type. The volume was subsequently converted to grams of ethanol, and values for each beverage type were added. The ethanol contents were calculated based on reported frequency, type of drink, and amount consumed, using the following alcohol content by volume reported by our patients and typically seen in China: 12% for beer and wine and 50% for Chinese liquor [10, 15, 16]. Alcohol consumption was quantified as grams per week (g/week). Among drinkers, men were grouped into three consumption categories (< 140, 140–419 and 420+ g per week) and women into three categories (< 70, 70–139 and 140+ g per week), broadly based on the recommended cutoffs for alcohol categories by the World Health Organization (WHO) and national drinking guidelines [15, 17]. Gevin the potential impact that alcohol addiction could have on cognitive function, psychiatric symptoms, and motor impairments, we conducted a comprehensive assessment of patients based on diagnostic criteria to ensure that all patients included in the analysis did not have alcohol addiction [18].

Demographic details, such as age, age at onset and duration of illness, were recorded. The symptom severity and disease stage were rated using the PSP Rating Scale (PSPRS), the Unified Parkinson's Disease Rating Scale (UPDRS), Freezing of Gait Questionnaire (FOG‐Q), and Hoehn and Yahr scale (H&Y). The Montreal Cognitive Assessment Scale (MoCA) and Mini Mental State Examination (MMSE) were used to assess cognitive function. The Hamilton Anxiety Rating Scale (HAM‐A) and Hamilton Depression Rating Scale (HAM‐D) were administered to estimate the presence and severity of anxiety and depression, respectively. The severity and frequency of nonmotor symptoms (NMSs) were assessed by using the validated NMS Scale (NMSS).

### 2.3 Statistical Analysis

Descriptive analysis provides summary statistics for the data, including mean ± standard deviation (SD) for continuous variables that are normally distributed, as determined by the Shapiro–Wilk test. For continuous variables that do not exhibit a normal distribution, the median and interquartile range (IQR, denoted as P25, P75) are provided. Additionally, for categorical variables, the frequencies and percentages (%) are reported. Quantitative variables were compared using the *t*‐test or the Mann–Whitney *U* test. Qualitative variables were compared using the chi‐squared test or fisher test. To assess variations in participant characteristics based on different levels of alcohol consumption, categorical variables were compared using the Pearson's chi‐squared test, while continuous variables were subjected to either one‐way ANOVA or Kruskal–Wallis test for comparison. For those variables that showed statistical significance, post hoc tests were conducted to further investigate the differences. Conducting multivariable linear regression to assess the associations between alcohol weekly consumption (light, moderate, or heavy) with motor and nonmotor symptoms. Model 1 was an unadjusted model without covariates. Model 2 adjusted for demographic variables, including age, gender, education level, and BMI. Model 3 adjusted for hypertension, diabetes mellitus, and smoking status, in addition to the variables that were already included in Model 2. Model 4 was a fully adjusted model, incorporating demographic variables, comorbidities, smoking status, disease duration, and phenotype. All statistical analyses were performed with R 4.4.1 software (R Project for Statistical Computing; http://www.r‐project.org). All the statistical tests were two‐sided, and differences were considered to be statistically significant when the *p* < 0.05.

## 3 Results

### 3.1 Clinical Variables in Whole PSP Samples

The demographic information is presented in Table 1. A total of 128 individuals with PSP were included, with a median age of 66.00 years. The overall prevalence of alcohol consumption was 45.31% (58 individuals) in this cohort. More than 90% of them were male, and 53.4% were classified as heavy drinkers. There was a statistically significant difference in education levels (*p* = 0.039) and smoking history (*p* < 0.001) between drinkers and nondrinkers. Furthermore, the PSPRS subtype was more prevalent among drinkers than among nondrinkers. No discernible distinctions were observed in relation to age at recruitment, age at disease onset, disease duration, or history of comorbidity.

**TABLE 1:** Baseline demographic and clinical characteristics of participants.

Characteristic	All patients (*N* = 128)	Drinker (*N* = 58)	Nondrinker (*N* = 70)	*p* value^a^
**Demographic characteristics**				
Gender (male, %)	76 (59.4)	53 (91.4)	23 (32.9)	**< 0.001**
Age at recruitment (years)	66.00 [61.00, 70.00]	67.00 [62.25, 71.00]	66.00 [61.00, 70.00]	0.352
Age at disease onset (years)	61.99 (6.59)	62.50 (6.43)	61.57 (6.73)	0.430
Disease duration (years)	3.00 [2.00, 4.00]	4.00 [3.00, 4.00]	3.00 [2.00, 4.00]	0.148
Body mass index (kg/m2)	24.44 (2.41)	24.60 (2.50)	24.32 (2.35)	0.517
Education(years)	9.00 [6.00, 12.00]	9.00 [6.00, 12.00]	6.00 [6.00, 9.00]	**0.039**
Subtype (yes, %)				
PSPRS	71 (55.5)	36 (62.1)	35 (50.0)	0.339
PSP‐P	24 (18.8)	9 (15.5)	15 (21.4)	
PSP‐PGF	24 (18.8)	11 (19.0)	13 (18.6)	
vPSP	9 (7.0)	2 (3.4)	7 (10.0)	
Smoking history (yes, %)	45 (35.2)	40 (69.0)	5 (7.1)	**< 0.001**
Drinking history (yes, %)	58 (45.31)	—	—	—
Light drinker	8 (6.25)	8 (13.8)	—	—
Moderate drinker	19 (14.84)	19 (32.8)	—	—
Heavy drinker	31 (24.22)	31 (53.4)	—	—
Drinking years (years)	0.00 [0.00, 30.00]	30.00 [30.00, 40.00]	—	—
Comorbidity (yes, %)				
Hypertension	50 (39.1)	21 (36.2)	29 (41.4)	0.674
Diabetes mellitus	21 (16.4)	8 (13.8)	13 (18.6)	0.626
**Motor symptoms**				
PSPRS	36.62 (11.22)	39.31 (11.34)	34.39 (10.69)	**0.013**
History	7.00 [5.00, 9.25]	7.50 [5.25, 9.00]	7.00 [5.00, 10.00]	0.812
Mentation	2.00 [1.00, 4.00]	3.00 [1.25, 5.75]	2.00 [1.00, 4.00]	**0.035**
Bulbar	3.00 [2.00, 4.00]	3.00 [2.25, 5.00]	3.00 [2.00, 4.00]	**0.033**
Oculomotor	7.50 [4.00, 10.00]	8.00 [5.25, 10.00]	7.00 [4.00, 9.75]	0.225
Limb motor	6.50 [4.00, 9.00]	7.00 [5.00, 10.00]	5.50 [3.25, 9.00]	0.051
Gait and midline	8.88 (2.88)	9.36 (2.92)	8.47 (2.80)	0.082
UPDRS total score	57.50 [44.75, 75.00]	59.50 [50.25, 77.25]	53.50 [40.75, 72.75]	**0.04**
UPDRS‐I	11.00 [7.00, 16.00]	10.00 [7.00, 16.00]	11.00 [7.00, 15.75]	0.848
UPDRS‐II	17.50 [13.00, 23.00]	19.00 [15.25, 23.00]	16.00 [12.25, 22.75]	**0.026**
UPDRS‐III	28.00 [21.00, 37.25]	30.00 [22.25, 37.75]	27.00 [18.00, 34.50]	**0.118**
FOG‐Q	14.00 [5.00, 19.25]	14.50 [10.00, 19.50]	12.50 [3.25, 19.00]	0.420
H&Y Stage	3.00 [3.00, 4.00]	3.00 [3.00, 4.00]	3.00 [3.00, 3.75]	0.318
**Cognitive function**				
MoCA	16.00 [11.75, 20.00]	16.00 [11.00, 19.00]	17.00 [12.00, 20.75]	**0.029**
Visuospatial/executive	1.00 [1.00, 3.00]	1.00 [1.00, 2.00]	2.00 [1.00, 3.00]	**0.001**
Naming	3.00 [2.00, 3.00]	3.00 [2.00, 3.00]	3.00 [2.00, 3.00]	0.820
Attention	4.00 [3.00, 5.00]	4.00 [3.00, 5.00]	4.00 [2.00, 5.00]	0.555
Language	1.00 [0.00, 2.00]	1.00 [0.00, 1.00]	1.00 [1.00, 2.00]	**0.011**
Abstraction	1.00 [1.00, 2.00]	1.00 [0.00, 2.00]	1.00 [1.00, 1.00]	0.516
Memory	1.00 [0.00, 2.00]	1.00 [0.00, 2.00]	1.00 [1.00, 2.00]	**0.021**
Orientation	5.00 [4.00, 5.00]	4.00 [2.25, 5.00]	5.00 [4.00, 6.00]	**0.011**
MMSE	21.00 [16.00, 24.00]	19.50 [16.00, 23.00]	21.00 [17.00, 25.00]	**0.049**
Orientation	7.00 [6.00, 8.00]	7.00 [5.00, 8.00]	7.00 [6.00, 9.00]	**0.010**
Registration	3.00 [2.00, 3.00]	3.00 [2.00, 3.00]	3.00 [2.00, 3.00]	0.566
Attention/calculation	3.00 [1.00, 4.00]	3.00 [1.00, 4.75]	2.50 [1.00, 4.00]	0.579
Recall	2.00 [1.00, 2.00]	2.00 [1.00, 3.00]	2.00 [1.00, 2.00]	0.950
Language	6.00 [5.00, 7.00]	5.00 [4.00, 6.00]	7.00 [5.25, 8.00]	**< 0.001**
Visuospatial	0.00 [0.00, 1.00]	0.00 [0.00, 0.75]	0.00 [0.00, 1.00]	0.305
**Nonmotor symptoms**				
NMSS‐total score	52.00 [36.00, 83.00]	52.50 [38.50, 84.00]	51.00 [31.50, 80.00]	0.428
Cardiovascular	0.00 [0.00, 1.00]	0.00 [0.00, 0.00]	0.00 [0.00, 1.00]	0.071
Sleep	10.00 [5.00, 19.00]	10.00 [5.00, 18.50]	9.50 [5.25, 18.75]	0.983
Mood	9.00 [1.00, 18.00]	9.00 [1.00, 18.00]	9.00 [1.25, 20.00]	0.582
Perceptual	0.00 [0.00, 1.00]	0.00 [0.00, 0.00]	0.00 [0.00, 1.00]	0.909
Attention	6.00 [2.00, 12.00]	7.50 [3.00, 15.00]	4.00 [1.00, 8.75]	**0.002**
Gastrointestinal	7.00 [2.00, 13.00]	8.00 [4.00, 14.00]	6.00 [2.00, 13.00]	0.237
Urinary	8.00 [0.75, 16.00]	7.00 [1.25, 16.00]	8.00 [0.00, 16.00]	0.938
Sexual	0.00 [0.00, 0.00]	0.00 [0.00, 0.00]	0.00 [0.00, 0.00]	0.545
Miscellaneous	1.00 [0.00, 6.00]	1.00 [0.00, 6.00]	1.00 [0.00, 4.00]	0.473
HAH‐D	12.41 (6.49)	11.81 (6.22)	12.90 (6.71)	0.346
HAM‐A	10.00 [6.00, 16.00]	10.00 [7.00, 16.00]	11.00 [6.00, 16.00]	0.973

^a^ Continuous variables are shown as mean (standard deviation) or as median (interquartile range [P25–P75]). Categorical variables are shown as frequency (percent). Group comparison in continuous variables was performed using Student's *t* test and Mann–Whitney *U* test. Chi‐squared test and Fisher's exact test were used for categorical variables. Significant difference was indicated in bold.

Table 1 illustrates the clinical variables, including both motor and nonmotor scores. Objective evaluations revealed that drinkers suffered from worse motor symptoms and cognitive performance than nondrinkers. In this population, drinkers had higher PSPRS scores (*p* = 0.013), primarily in mentation (*p* = 0.035) and bulbar (0.033). Drinkers' UPDRS scores also achieved higher overall, as did UPDRS‐II and UPDRS‐III scores, indicating worse motor symptoms. Patients with a history of drinking displayed poorer levels of cognitive function as measured by the MOCA and MMSE: they mainly had impaired visuospatial/executive functions (*p* = 0.001), language (*p* = 0.011), memory (*p* = 0.021), and orientation (*p* = 0.011) domains in the MOCA, while in the orientation (*p* = 0.010) and language (*p* < 0.001) domains in the MMSE. Although the total NMSS scores did not significantly differ between the two groups, there was a disparity between the two groups in terms of the attention domain (*p* = 0.002). In addition, FOG‐Q, H&Y, HAM‐A, and HAM‐D were not significantly different between the two groups.

### 3.2 Clinical Variables in Male Patients

A significant portion of individuals diagnosed with PSP who had a history of drinking were male. We therefore analyzed the differences in clinical symptoms between drinkers and nondrinkers in male PSP patients to rule out the effect of gender differences (Table 2). The results showed that a higher proportion of men who drink alcohol were PSPRS phenotype (62.3%). Even, they have a worse eye movement (oculomotor subscales of the PSPRS, *p* = 0.024) and limb motor (*p* = 0.043). The results from the NMSS showed that male drinkers were more likely to experience dizziness (cardiovascular subscales, *p* = 0.023). The remaining results are generally consistent with those obtained when comparing drinkers and nondrinkers in the total patient population.

**TABLE 2:** Baseline demographic and clinical characteristics of male participants.

Characteristic	All male patients (*N* = 76)	Drinker (*N* = 53)	Nondrinker (*N* = 23)	*p* value^a^
**Demographic characteristics**				
Age at recruitment (years)	66.01 (6.96)	66.13 (6.51)	65.74 (8.06)	0.823
Age at disease onset (years)	62.39 (6.85)	62.42 (6.47)	62.35 (7.83)	0.969
Disease duration (years)	4.00 [3.00, 4.00]	4.00 [3.00, 4.00]	3.00 [2.50, 4.00]	0.544
Body mass index (kg/m^2^)	24.85 (2.42)	24.74 (2.53)	25.10 (2.17)	0.556
Education(years)	9.00 [6.00, 12.00]	9.00 [6.00, 12.00]	9.00 [6.00, 10.50]	0.171
Subtype (yes, %)				
PSPRS	43 (56.6)	33 (62.3)	10 (43.5)	0.299
PSP‐P	15 (19.7)	8 (15.1)	7 (30.4)	
PSP‐PGF	14 (18.4)	10 (18.9)	4 (17.4)	
vPSP	4 (5.3)	2 (3.8)	2 (8.7)	
Smoking history (yes, %)	42 (55.3)	37 (69.8)	5 (21.7)	< 0.001
Drinking history (yes, %)	53 (69.7)	53 (100)	—	—
Light drinker	7 (9.20)	7 (13.21)	—	—
Moderate drinker	19 (25.0)	19 (35.85)	—	—
Heavy drinker	27 (35.50)	27 (50.94)	—	—
Drinking years (years)	30.00 [0.00, 40.00]	30.00 [30.00, 40.00]	—	—
Comorbidity (yes, %)				
Hypertension	28 (36.8)	18 (34.0)	10 (43.5)	0.595
Diabetes mellitus	15 (19.7)	8 (15.1)	7 (30.4)	0.219
**Motor symptoms**				
PSPRS	37.09 (11.93)	39.79 (11.48)	30.87 (10.75)	**0.002**
History	7.00 [5.00, 9.00]	7.00 [5.00, 9.00]	6.00 [4.50, 7.50]	0.169
Mentation	2.00 [1.00, 4.00]	3.00 [1.00, 6.00]	1.00 [0.00, 3.00]	**0.013**
Bulbar	3.00 [2.00, 4.25]	3.00 [2.00, 5.00]	3.00 [2.00, 4.00]	0.055
Oculomotor	8.00 [4.00, 10.00]	9.00 [6.00, 10.00]	5.00 [3.00, 8.50]	**0.024**
Limb motor	7.05 (3.74)	7.62 (3.73)	5.74 (3.49)	**0.043**
Gait and midline	9.05 (3.19)	9.47 (2.95)	8.09 (3.58)	0.082
UPDRS total score	59.50 [46.25, 75.00]	60.00 [51.00, 80.00]	54.00 [36.00, 67.50]	**0.033**
UPDRS_I	11.45 (6.20)	12.02 (6.11)	10.13 (6.35)	0.225
UPDRS_II	18.50 [14.00, 25.25]	19.00 [15.00, 25.00]	15.00 [11.00, 25.50]	0.058
UPDRS_III	29.00 [22.00, 36.25]	30.00 [22.00, 38.00]	28.00 [16.50, 32.00]	0.103
FOG‐Q	13.50 [5.00, 20.00]	14.00 [10.00, 18.00]	11.00 [1.00, 22.00]	0.543
H&Y Stage	3.00 [3.00, 4.00]	3.00 [3.00, 4.00]	3.00 [3.00, 3.00]	0.17
**Cognitive function**				
MoCA	17.00 [11.00, 20.00]	16.00 [11.00, 20.00]	18.00 [15.50, 21.00]	**0.028**
Visuospatial/executive	1.00 [1.00, 2.00]	1.00 [1.00, 2.00]	2.00 [1.00, 3.00]	**0.011**
Naming	3.00 [2.00, 3.00]	3.00 [2.00, 3.00]	3.00 [2.00, 3.00]	0.672
Attention	4.00 [3.00, 5.00]	4.00 [3.00, 5.00]	4.00 [3.00, 5.50]	0.208
Language	1.00 [0.00, 2.00]	1.00 [0.00, 1.00]	1.00 [1.00, 2.00]	**0.044**
Abstraction	1.00 [1.00, 2.00]	1.00 [0.00, 2.00]	1.00 [1.00, 1.50]	0.775
Memory	1.00 [0.00, 2.00]	1.00 [0.00, 2.00]	2.00 [1.00, 2.00]	**0.041**
Orientation	5.00 [3.00, 5.00]	4.00 [3.00, 5.00]	5.00 [4.00, 5.50]	0.171
MMSE	21.00 [17.00, 24.00]	20.00 [16.00, 23.00]	23.00 [19.50, 25.00]	**0.014**
Orientation	7.00 [5.75, 8.00]	7.00 [5.00, 8.00]	8.00 [6.50, 9.00]	**0.012**
Registration	3.00 [2.00, 3.00]	3.00 [2.00, 3.00]	3.00 [3.00, 3.00]	0.464
Attention/Calculation	3.00 [1.00, 5.00]	3.00 [1.00, 5.00]	3.00 [2.00, 4.50]	0.703
Recall	2.00 [1.00, 3.00]	2.00 [1.00, 3.00]	2.00 [1.00, 2.50]	0.819
Language	6.00 [4.00, 7.00]	5.00 [4.00, 6.00]	7.00 [6.00, 8.00]	**< 0.001**
Visuospatial	0.00 [0.00, 1.00]	0.00 [0.00, 1.00]	0.00 [0.00, 1.00]	0.354
**Nonmotor symptoms**				
NMSS‐total score	51.00 [37.75, 80.50]	53.00 [38.00, 84.00]	46.00 [35.00, 59.00]	0.13
Cardiovascular	0.00 [0.00, 1.00]	0.00 [0.00, 0.00]	0.00 [0.00, 1.50]	**0.023**
Sleep	8.00 [4.00, 16.00]	9.00 [5.00, 17.00]	6.00 [4.00, 12.00]	0.475
Mood	8.50 [1.00, 14.25]	9.00 [1.00, 18.00]	3.00 [1.00, 10.00]	0.244
Perceptual	0.00 [0.00, 1.00]	0.00 [0.00, 0.00]	0.00 [0.00, 1.00]	0.358
Attention	6.00 [3.00, 12.00]	8.00 [3.00, 16.00]	3.00 [1.50, 6.00]	**0.001**
Gastrointestinal	8.00 [3.75, 14.00]	8.00 [4.00, 14.00]	7.00 [2.00, 12.50]	0.421
Urinary	6.50 [1.00, 16.00]	8.00 [1.00, 16.00]	3.00 [0.50, 17.00]	0.665
Sexual	0.00 [0.00, 0.00]	0.00 [0.00, 0.00]	0.00 [0.00, 0.00]	0.904
Miscellaneous	1.00 [0.00, 6.00]	1.00 [0.00, 6.00]	0.00 [0.00, 2.00]	0.573
HAH‐D	11.82 (6.75)	11.91 (6.33)	11.61 (7.77)	0.861
HAM‐A	11.28 (6.45)	11.32 (6.09)	11.17 (7.34)	0.928

^**a**^ Continuous variables are shown as mean (standard deviation) or as median (interquartile range). Categorical variables are shown as frequency (percent). Group comparison in continuous variables was performed using Student's *t* test and Mann–Whitney *U* test. Chi‐squared test and Fisher's exact test were used for categorical variables. Significant difference was indicated in bold.

### 3.3 Subgroup Analysis Based on Alcohol Consumption

**FIGURE 2:**
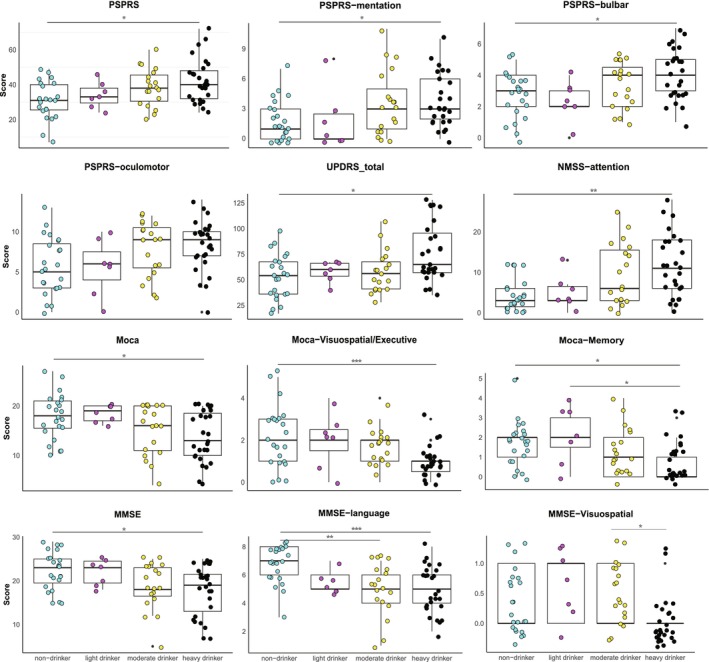
Clinical features of male PSP patients with different levels of alcohol consumption (nondrinker, light drinker, moderate drinker, and heavy drinker). Variables were compared among four groups by one‐way analysis of variance for normally distributed data or Kruskal–Wallis test for abnormally distributed data. *p* values of the posterior comparisons were adjusted by Bonferroni correction. *p < 0.05, **p < 0.01, ***p < 0.001.

We stratified male patients according to alcohol consumption and compared the clinical symptoms of nondrinkers with those of patients who drank alcohol in different amounts (Table 3 and Figure 2). No difference in years of drinking history between groups. According to the PSPRS, the results illustrated that motor symptoms were significantly worse in the heavy drinking group, especially mentation (*p =* 0.012), bulbar (*p* = 0.013), and oculomotor (*p* = 0.041). Upon comparing the groups based on UPDRS scores, it was observed that there was a difference in the total scores (*p* = 0.017); however, no statistically significant differences were found among the subitems. Simultaneously, there were no differences among the groups in terms of the FOG‐Q and H&Y. The results of the MOCA and MMSE suggest that heavy alcohol consumption impairs cognitive function, whereas light alcohol consumption may have a protective effect on cognitive function, although the difference was not statistically significant (Table 3 and Figure 2). The primary cognitive domains affected include visuospatial and executive abilities (subitem of MOCA, *p* = 0.001, subitem of MMSE, *p* = 0.007), memory (p = 0.007), and language (*p* < 0.001). The subitem attention of the NMSS revealed differences (*p* = 0.003), which also indicated variations in cognitive function among the groups. We also carried out a comprehensive grouped analysis on the entire patient population, with the results neatly summarized and presented in the Table S1 for easy reference.

**TABLE 3:** Comparison of severity of motor and nonmotor symptoms of male patients according to weekly alcohol intake.

Characteristic	Nondrinker (*N* = 23)	Light drinker (*N* = 7)	Moderate drinker (*N* = 19)	Heavy drinker (*N* = 27)	*p* value^a^
**Demographic characteristics**					
Age at recruitment (years)	65.74 (8.06)	67.29 (7.89)	68.05 (6.81)	64.48 (5.69)	0.366
Age at disease onset (years)	62.35 (7.83)	63.14 (7.69)	64.37 (6.73)	60.85 (5.76)	0.394
Disease duration (years)	3.00 [2.50, 4.00]	4.00 [3.50, 4.00]	4.00 [3.00, 4.00]	4.00 [3.00, 4.50]	0.867
Body mass index (kg/m^2^)	25.10 (2.17)	24.51 (2.92)	24.82 (2.11)	24.75 (2.78)	0.935
Education(years)	9.00 [6.00, 10.50]	6.00 [6.00, 10.50]	6.00 [6.00, 9.00]	12.00 [9.00, 12.00]	**0.006**
Subtype (yes, %)					
PSPRS	10 (43.5)	5 (71.4)	11 (57.9)	17 (63.0)	0.894
PSP‐P	7 (30.4)	1 (14.3)	3 (15.8)	4 (14.8)	
PSP‐PGF	4 (17.4)	1 (14.3)	4 (21.1)	5 (18.5)	
vPSP	2 (8.7)	0 (0.0)	1 (5.3)	1 (3.7)	
Smoking history (yes, %)	5 (21.7)	4 (57.1)	13 (68.4)	20 (74.1)	**0.001**
Drinking years (years)	0.00 [0.00, 0.00]	30.00 [30.00, 35.00]	40.00 [30.00, 40.00]	30.00 [30.00, 40.00]	**< 0.001**
Comorbidity (yes, %)	—				
Hypertension	10 (43.5)	4 (57.1)	3 (15.8)	11 (40.7)	0.14
Diabetes mellitus	7 (30.4)	2 (28.6)	4 (21.1)	2 (7.4)	0.202
**Motor symptoms**					
PSPRS	30.87 (10.75)	34.00 (7.51)	38.42 (10.75)	42.26 (12.41)	**0.005**
History	6.00 [4.50, 7.50]	8.00 [7.50, 8.50]	7.00 [5.00, 8.50]	7.00 [5.50, 10.00]	0.469
Mentation	1.00 [0.00, 3.00]	0.00 [0.00, 2.50]	3.00 [1.00, 5.00]	3.00 [2.00, 6.00]	**0.012**
Bulbar	3.00 [2.00, 4.00]	2.00 [2.00, 3.00]	4.00 [2.00, 4.50]	4.00 [3.00, 5.00]	**0.013**
Oculomotor	5.00 [3.00, 8.50]	6.00 [4.00, 7.50]	9.00 [5.50, 10.50]	9.00 [7.00, 10.00]	**0.041**
Limb motor	5.74 (3.49)	7.43 (3.05)	7.11 (2.88)	8.04 (4.42)	0.189
Gait and midline	8.09 (3.58)	8.29 (2.36)	8.84 (2.17)	10.22 (3.40)	0.099
UPDRS Total Score	54.00 [36.00, 67.50]	60.00 [53.50, 66.00]	56.00 [41.00, 67.50]	65.00 [57.00, 95.50]	**0.017**
UPDRS‐I	10.13 (6.35)	12.71 (4.23)	11.47 (5.95)	12.22 (6.76)	0.634
UPDRS‐II	15.00 [11.00, 25.50]	20.86 (5.96)	18.58 (8.18)	23.15 (11.08)	0.206
UPDRS‐III	28.00 [16.50, 32.00]	30.00 [22.00, 33.00]	24.00 [21.50, 37.50]	32.00 [26.00, 50.00]	0.077
FOG‐Q	11.00 [1.00, 22.00]	12.00 [8.50, 12.50]	13.00 [10.00, 18.00]	16.00 [11.50, 20.00]	0.365
H&Y Stage	3.00 [3.00, 3.00]	3.00 [3.00, 4.00]	3.00 [3.00, 3.00]	3.00 [3.00, 4.00]	0.205
**Cognitive function**					
MoCA	18.00 [15.50, 21.00]	19.00 [17.00, 20.00]	16.00 [11.00, 20.00]	13.00 [10.00, 18.50]	**0.025**
Visuospatial/Executive	2.00 [1.00, 3.00]	2.00 [1.50, 2.50]	2.00 [1.00, 2.00]	1.00 [0.50, 1.00]	**0.001**
Naming	3.00 [2.00, 3.00]	3.00 [3.00, 3.00]	2.00 [2.00, 3.00]	3.00 [2.00, 3.00]	0.255
Attention	4.00 [3.00, 5.50]	5.00 [3.50, 5.00]	4.00 [2.50, 5.00]	4.00 [3.00, 5.00]	0.434
Language	1.00 [1.00, 2.00]	1.00 [1.00, 1.50]	1.00 [0.00, 1.00]	1.00 [0.00, 1.00]	0.15
Abstraction	1.00 [1.00, 1.50]	1.00 [0.50, 1.50]	1.00 [0.50, 2.00]	1.00 [0.00, 2.00]	0.83
Memory	2.00 [1.00, 2.00]	2.00 [1.50, 3.00]	1.00 [0.00, 2.00]	0.00 [0.00, 1.00]	**0.007**
Orientation	5.00 [4.00, 5.50]	4.00 [4.00, 6.00]	5.00 [2.00, 5.00]	4.00 [2.50, 5.00]	0.29
MMSE	23.00 [19.50, 25.00]	23.00 [19.50, 24.50]	18.00 [16.50, 23.00]	19.00 [13.00, 21.50]	**0.027**
Orientation	8.00 [6.50, 9.00]	7.00 [6.50, 8.00]	7.00 [4.50, 7.50]	7.00 [5.00, 7.50]	0.056
Registration	3.00 [3.00, 3.00]	3.00 [3.00, 3.00]	3.00 [2.00, 3.00]	3.00 [2.00, 3.00]	0.667
Attention/Calculation	3.00 [2.00, 4.50]	5.00 [4.00, 5.00]	2.00 [1.00, 4.00]	3.00 [1.00, 4.00]	0.11
Recall	2.00 [1.00, 2.50]	1.00 [1.00, 2.00]	2.00 [1.50, 3.00]	1.00 [0.50, 2.50]	0.35
Language	7.00 [6.00, 8.00]	5.00 [5.00, 6.00]	5.00 [4.00, 6.00]	5.00 [4.00, 6.00]	**< 0.001**
Visuospatial	0.00 [0.00, 1.00]	1.00 [0.00, 1.00]	0.00 [0.00, 1.00]	0.00 [0.00, 0.00]	**0.007**
**Nonmotor symptoms**					
NMSS‐total score	46.00 [35.00, 59.00]	58.00 [50.00, 74.50]	44.00 [36.50, 62.50]	65.00 [40.00, 98.00]	0.162
Cardiovascular	0.00 [0.00, 1.50]	0.00 [0.00, 0.50]	0.00 [0.00, 0.00]	0.00 [0.00, 0.00]	0.145
Sleep	6.00 [4.00, 12.00]	19.00 [11.50, 23.50]	9.00 [6.00, 12.00]	8.00 [2.00, 18.00]	0.372
Mood	3.00 [1.00, 10.00]	1.00 [0.00, 20.50]	7.00 [0.50, 13.50]	12.00 [2.00, 18.00]	0.357
Perceptual	0.00 [0.00, 1.00]	0.00 [0.00, 0.00]	0.00 [0.00, 0.00]	0.00 [0.00, 1.50]	0.518
Attention	3.00 [1.50, 6.00]	3.00 [3.00, 6.50]	6.00 [3.00, 15.50]	11.00 [6.00, 18.00]	**0.003**
Gastrointestinal	7.00 [2.00, 12.50]	4.00 [3.00, 4.50]	6.00 [4.00, 14.00]	12.00 [7.00, 14.50]	0.124
Urinary	3.00 [0.50, 17.00]	2.00 [2.00, 19.50]	8.00 [2.00, 13.00]	6.00 [0.50, 19.50]	0.977
Sexual	0.00 [0.00, 0.00]	0.00 [0.00, 0.00]	0.00 [0.00, 0.00]	0.00 [0.00, 0.00]	0.786
Miscellaneous	0.00 [0.00, 2.00]	0.00 [0.00, 3.50]	0.00 [0.00, 4.00]	1.00 [0.00, 9.50]	0.515
HAH‐D	11.61 (7.77)	14.00 (10.03)	11.47 (5.45)	11.67 (5.92)	0.851
HAM‐A	11.17 (7.34)	13.43 (6.29)	10.84 (6.13)	11.11 (6.14)	0.834

^**a**^ Continuous variables are shown as mean (standard deviation) or as median (interquartile range [P25–P75]). Categorical variables are shown as frequency (percent). Group comparison in continuous variables was performed using Student's t test and Mann–Whitney *U* test. Chi‐squared test and Fisher's exact test were used for categorical variables. Significant difference was indicated in bold.

### 3.4 Relationship Between Alcohol Consumption and Clinical Symptoms

Table 4 describes the association between alcohol consumption and clinical symptoms of male patients, conducting trend tests on four groups (nondrinker, light drinker, moderate drinker, and heavy drinker) using a multivariate linear regression analysis. After controlling for all confounding factors, heavy drinkers, in comparison with nondrinkers, exhibited significantly higher scores for PSPRS (*β* = 13.516, 95% CI = 5.892–21.139) and UPDRS (*β* = 27.894, 95% CI = 11.511–44.278) as well as significantly lower MOCA scores (*β* = −6.249, 95% CI = −9.134 to −3.364) and MMSE scores (*β* = −6.257, 95% CI = ‐9.196 to −3.318). The PSPRS scores in the moderate alcohol consumption group also exhibited significant differences. However, no significant differences were found between light drinkers and nondrinkers. In the unadjusted Model 1 and Model 2, we did not observe a rising trend in scores for PSPRS in moderate drinkers. After adjusting for comorbidity and smoking history (Model 3) and disease‐related factors (disease duration and phenotype, Model 4), this association became significant (*p* < 0.05) (Table S2).

**TABLE 4:** Association between alcohol consumption and motor symptoms and cognitive functions in male patients.

	Model 1		Model 2		Model 3		Model 4	
	*β* (95%CI)	*p*	β (95%CI)	*p*	*β* (95%CI)	*p*	*β* (95%CI)	*p*
**PSPRS**								
Nondrinker	0 (Ref)		0 (Ref)		0 (Ref)		0 (Ref)	
Light drinker	3.13 (−6.471, 12.732)	0.518	2.9 (−6.831, 12.631)	0.554	4.244 (−5.809, 14.297)	0.402	2.475 (−7.437, 12.388)	0.620
Moderate drinker	7.551 (0.656, 14.447)	**0.032**	7.09 (0.072, 14.108)	**0.048**	8.975 (1.231, 16.719)	**0.024**	8.949 (1.401, 16.497)	**0.021**
Heavy drinker	11.39 (5.078, 17.701)	**< 0.001**	11.566 (4.855, 18.277)	**< 0.001**	14.287 (6.532, 22.043)	**< 0.001**	13.516 (5.892, 21.139)	**< 0.001**
**UPDRS**								
Nondrinker	0 (Ref)		0 (Ref)		0 (Ref)		0 (Ref)	
Light drinker	5.087 (−15.082, 25.256)	0.617	5.389 (−15.019, 25.796)	0.6	6.534 (−14.58, 27.649)	0.5388	3.943 (−17.36, 25.245)	0.7128
Moderate drinker	4.824 (−9.661, 19.309)	0.509	4.207 (−10.511, 18.924)	0.570	8.573 (−7.691, 24.837)	0.2964	8.051 (−8.17, 24.272)	0.3251
Heavy drinker	24.124 (10.866, 37.382)	**< 0.001**	26.159 (12.085, 40.232)	**< 0.001**	29.735 (13.447, 46.023)	**< 0.001**	27.894 (11.511, 44.278)	**0.0012**
**Moca**								
Nondrinker	0 (Ref)		0 (Ref)		0 (Ref)		0 (Ref)	
Light drinker	0.559 (−3.501, 4.619)	0.785	0.824 (−2.757, 4.405)	0.648	0.336 (−3.379, 4.051)	0.857	0.472 (−3.279, 4.224)	0.802
Moderate drinker	−2.712 (−5.627, 0.204)	0.068	−2.006 (−4.589, 0.577)	0.126	−2.279 (−5.141, 0.583)	0.117	−2.373 (−5.23, 0.484)	0.102
Heavy drinker	−4.166 (−6.834, −1.497)	**0.003**	−5.57 (−8.04, −3.101)	**< 0.001**	−6.168 (−9.034, −3.302)	**< 0.001**	−6.249 (−9.134, −3.364)	**< 0.001**
**MMSE**								
Nondrinker	0 (Ref)		0 (Ref)		0 (Ref)		0 (Ref)	
Light drinker	−0.217 (−4.526, 4.091)	0.920	0.135 (−3.575, 3.846)	0.942	−0.088 (−3.894, 3.719)	0.964	0.121 (−3.701, 3.942)	0.950
Moderate drinker	−3.428 (−6.522, −0.333)	**0.030**	−2.662 (−5.339, 0.014)	0.051	−2.342 (−5.274, 0.59)	0.116	−2.441 (−5.351, 0.469)	0.097
Heavy drinker	−4.403 (−7.235, −1.57)	**0.003**	−5.959 (−8.518, −3.4)	**< 0.001**	−6.198 (−9.134, −3.262)	**< 0.001**	−6.257 (−9.196, −3.318)	**< 0.001**

*Note:* Model 1: No adjustment; Model 2: Adjusted for demographic variables (age, gender, education level, and BMI); Model 3: Adjusted as in Model 2, additionally adjusted for hypertension, diabetes mellitus, and smoking status; Model 4: Adjusted as in Model 3, additionally adjusted for duration and phenotype.

## 4 Discussion

An evaluative analysis of PSP patients conducted at our center has revealed that alcohol consumption exacerbates clinical symptoms, as assessed by the PSPRS and UPDRS. Additionally, cognitive decline, particularly in visuospatial and executive abilities, memory, and language, was worsened by alcohol consumption, as indicated by the MOCA and MMSE. Given that PSP predominantly affects males and the majority of alcohol consumers in our cohort were male, we performed gender stratification to eliminate any potential gender‐related bias [19, 20, 21]. After stratification, we found that, among male patients, alcohol continued to exacerbate these symptoms.

Research indicates that prolonged exposure to plants of the Anacardiaceae family, particularly anacardic acid which can disrupt mitochondrial complex I, significantly increases the risk of PSP [22, 23, 24, 25, 26]. A case–control study revealed that PSP patients consumed meat or poultry more frequently than controls [12] and fresh produce and reduced red meat intake may impact PSP‐related dementia risk [27, 28]. All of the aforementioned findings collectively indicate that dietary factors play a significant role in the development of PSP. A 2021 US study found overlapping genes between alcohol use disorder and neurodegenerative diseases, suggesting interventions for alcohol issues might benefit neurodegenerative diseases [29]. Given alcohol's role in Chinese social interactions, studying its effects on PSP patients is crucial.

PSP is a neurodegenerative disease that can be influenced by various factors, such as environmental factors, neuroinflammation, and neural immunity [3]. Alcohol consumption exacerbates PSP symptoms, akin to its effects on Alzheimer's disease (AD), both being Tauopathies. In AD patients with alcohol use disorder, alcohol disrupts intestinal microbiota imbalance [30]. A Norway study suggested that alcohol‐dependent AD patients with intestinal permeability and microbiota imbalance had reduced social abilities [31]. Transplanting the intestinal microbiota of AD patients into mice exacerbates AD symptoms, including anxiety, depression, and cognitive decline [32]. In mouse studies, transplantation of the fecal microbiota from AD patients after antibiotic and polyethylene glycol treatment replicated changes in social behavior and depressive‐like symptoms, causing demyelination, neurotransmission impairment, and inflammation [31]. Research from Zhengzhou University's Affiliated Hospital indicated that PSP patients exhibit imbalances in their gut microbiota, and assessing the status of the gut microbiota could serve as an auxiliary diagnostic criterion for PSP. Fecal microbiota transplantation may alleviate some symptoms in PSP patients [33].

Literature confirms that astrocytes in the cerebellar region of mice can metabolize ethanol into acetic acid, influencing the balance and coordination [34]. Knocking out key enzymes involved in ethanol metabolism normalizes acetic acid levels and restores these functions. We hypothesize that in PSP, when exposed to heavy alcohol consumption, cortical astrocytes overproduce acetic acid, which neurons absorb, impairing function and exacerbating motor disorders. From another perspective, alcohol can trigger inflammatory reactions in brain microglia, potentially leading to neuronal death [35]. Alcohol use disorders in PD patients can cause neuroinflammation, which may exacerbate motor symptoms [5]. Autopsy results in elevated expression of inflammation‐related factors in the brain in PSP patients [36], and excessive alcohol consumption may promote the progression of PSP motor disorders by exacerbating neuroinflammation. Furthermore, research on AD indicates that alcohol and its metabolite, acetaldehyde, exert direct neurotoxic effects, causing lasting damage to brain structure and function [37].

Our data analysis revealed that alcohol consumption has an impact on the cognitive functions of PSP patients. Studies in the United States and France have linked frequent and heavy alcohol consumption to early onset and faster progression of AD and increased dementia risk among European populations, respectively [38, 39]. Further investigation revealed that hippocampal atrophy in heavy drinking AD patients may be the underlying cause of their cognitive decline [10]. In our study, based on the subitem of cognitive analysis, the most severe impairment was observed in visualspatial function. Previous literature has reported that individuals with alcohol use disorder may experience difficulties in visualspatial function, potentially attributed to cortical involvement [40]. Given the limitations in patient numbers and incomplete imaging data, it is conceivable that the PSP patients who consume alcohol may experience more severe cortical atrophy, leading to more pronounced cognitive impairments. On the other hand, eye movements are typically restricted in the majority of PSP patients, and excessive alcohol consumption exacerbates oculomotor deficits, potentially serving as another crucial factor contributing to the impairment of visuospatial function in this condition.

Not all cognitive effects associated with alcohol consumption are detrimental. Multiple studies have indicated that light to moderate alcohol consumption appears to decrease the risk of dementia and cognitive decline [41, 42, 43]. Research involving 19,887 elderly Americans demonstrated a U‐shaped relationship between weekly alcohol intake and cognitive function with Optimal cognitive performance observed at 10–14 drinks per week [44]. In our stratified analysis based on alcohol consumption, we categorized patients into high, moderate, and light drinking groups. Our findings indicate that low levels of alcohol consumption may exert a protective influence on cognitive function, albeit without achieving statistical significance in the results. However, the heavy drinking group exhibited definite cognitive impairment. We hypothesize that alcohol‐related problems experienced by our patients may stem from episodic heavy drinking or chronic alcohol abuse, often driven by work or social stress, rather than the regular low‐volume drinking reported in the literature.

This study has several limitations. First, the sample size was relatively small, and all participants were Chinese, which reduces the generalizability of our conclusions due to the homogeneity of the population. Second, the diagnosis was based on clinical criteria because there were no pathology‐proven cases available for this study, but patients who met the clinical criteria were given a diagnosis of possible or probable PSP with relatively high specificity. Third, a notable limitation of our study is the absence of more detailed neuropsychological assessment and imaging data. A comprehensive neuropsychological evaluation would have provided deeper insights into the cognitive dysfunction of the patients, while imaging data could have offered valuable information about structural and functional brain abnormalities. We collected imaging data from a subset of patients, but the small number and lack of follow‐up data further limits our study. Long‐term follow‐up is indispensable for determining the enduring effects of alcohol on the progression of PSP. This limitation hampers our ability to fully comprehend the cognitive domain impairments linked to alcohol consumption and the underlying neural mechanisms. Future studies should endeavor to incorporate these assessments to gain a more comprehensive understanding of the relationship between neuropsychological status and brain function in PSP patients who consume alcohol.

## 5 Conclusion

Our findings indicate an association between excessive alcohol consumption and more pronounced symptoms of PSP, especially cognitive dysfunction, which raises the possibility that alcohol intake may play a role in modulating the clinical course of the disease. However, taking into account potential confounding variables, this observation is preliminary and further research is necessary to definitively establish a causal link between alcohol intake and PSP progression.

## Author Contributions

**Min Tian:** study conception and design, investigation, formal analysis, data curation, writing the original draft. **Min Tian, Bohan Zhang, Heyin Liu, Yinlian Han, Mu Yang, Ruonan Duan, Dandan Guo, Chengyuan Song, Jun Ma, Yiming Liu:** material preparation and data collection. **Yiming Liu:** study conception and design, investigation, writing – review and editing. All authors contributed to the article and approved the submitted version.

## Ethics Statement

All procedures performed in studies involving human participants were in accordance with the ethical standards of the institutional and/or national research committee and with the 1975 Helsinki Declaration and its later amendments or comparable ethical standards. Informed consent was obtained from all the patients included in the study. The study was approved by the Ethics Committee of Qilu Hospital of Shandong University (KYLL‐202008‐122‐3).

## Conflicts of Interest

The authors declare no conflicts of interest.

## Supporting information


Table S1.
Table S2.

## Data Availability

The data supporting the findings of this study are available within the article and its Supporting Information. Further inquiries can be directed to the corresponding author.
