# Supplementary material for: The Effect of Alcohol Consumption on Progressive Supranuclear Palsy: A Cross‐Sectional Study
Source: CNS Neurosci Ther. 2024 Dec 23;30(12):e70146. doi: 10.1111/cns.70146 (PMC11664458; doi:10.1111/cns.70146)
Supplement: Supplementary file 1 — Table S1. Table S2. [file CNS-30-e70146-s001.docx]

**Supplementary Table 1 Comparison of severity of motor and non-motor symptoms of patients according to weekly alcohol intake**

| **Characteristic** | **Non-drinker**  (N = 70) | | | **Light drinker**  (N=8) | | **Moderate drinker**  (N=19) | | **Heavy drinker**  (N=31) | **p value^*^** |
| --- | --- | --- | --- | --- | --- | --- | --- | --- | --- |
| **Demographic characteristics** |  | | |  | |  | |  |  |
| Gender (male, %) | | 23 (32.9) | 7 (87.5) | | 19 (100) | | 27 (87.1) | | **<0.001** |
| Age at recruitment (years) | | 66.00 [61.00, 70.00] | 66.00 [62.50, 70.50] | | 70.00 [65.00, 71.50] | | 65.00 [61.50, 70.00] | | 0.167 |
| Age at disease onset (years) | | 61.57 (6.73) | 63.25 (7.13) | | 64.37 (6.73) | | 61.16 (5.94) | | 0.318 |
| Disease duration (years) | | 3.00 [2.00, 4.00] | 4.00 [3.00, 4.00] | | 4.00 [3.00, 4.00] | | 3.00 [2.50, 4.50] | | 0.45 |
| Body mass index (kg/m2) | | 24.32 (2.35) | 24.19 (2.85) | | 24.82 (2.11) | | 24.56 (2.68) | | 0.849 |
| Education(years) | | 6.00 [6.00, 9.00] | 6.00 [6.00, 9.75] | | 6.00 [6.00, 9.00] | | 12.00 [7.50, 12.00] | | **0.017** |
| Subtype (yes, %) | |  |  | |  | |  | | 0.9 |
| PSP-RS | | 35 (50.0) | 6 (75.0) | | 11 (57.9) | | 19 (61.3) | |  |
| PSP-P | | 15 (21.4) | 1 (12.5) | | 3 (15.8) | | 5 (16.1) | |  |
| PSP-PGF | | 13 (18.6) | 1 (12.5) | | 4 (21.1) | | 6 (19.4) | |  |
| vPSP | | 7 (10.0) | 0 (0.0) | | 1 (5.3) | | 1 (3.2) | |  |
| Smoking history (yes, %) | | 5 (7.1) | 4 (50.0) | | 13 (68.4) | | 23 (74.2) | | **<0.001** |
| Drinking years (years) | | 0.00 [0.00, 0.00] | 30.00 [27.50, 32.50] | | 40.00 [30.00, 40.00] | | 30.00 [30.00, 40.00] | | **-** |
| Comorbidity (yes, %) | | - |  | |  | |  | |  |
| Hypertension | | 29 (41.4) | 4 (50.0) | | 3(15.8) | | 14 (45.2) | | 0.146 |
| Diabetes mellitus | | 13 (18.6) | 2 (25.0) | | 4 (21.1) | | 2 (6.5) | | 0.361 |
| **Motor symptoms** | |  |  | |  | |  | |  |
| PSPRS | | 34.39 (10.69) | 35.50 (8.14) | | 38.42 (10.75) | | 40.84 (12.36) | | **0.05** |
| History | | 7.00 [5.00, 10.00] | 8.00 [7.75, 8.25] | | 7.00 [5.00, 8.50] | | 7.00 [6.00, 10.00] | | 0.914 |
| Mentation | | 2.00 [1.00, 4.00] | 1.00 [0.00, 4.25] | | 3.00 [1.00, 5.00] | | 3.00 [2.00, 5.50] | | 0.082 |
| Bulbar | | 3.00 [2.00, 4.00] | 2.50 [2.00, 3.25] | | 3.00 [2.00, 4.50] | | 4.00 [3.00, 5.00] | | **0.029** |
| Oculomotor | | 7.00 [4.00, 9.75] | 6.00 [5.00, 8.25] | | 9.00 [5.50, 10.50] | | 8.00 [5.00, 10.00] | | 0.352 |
| Limb motor | | 5.50 [3.25, 9.00] | 7.50 [6.00, 8.25] | | 7.00 [5.50, 9.50] | | 8.00 [4.00, 11.00] | | 0.271 |
| Gait and midline | | 8.47 (2.80) | 8.50 (2.27) | | 8.84 (2.17) | | 9.9 (3.40) | | 0.14 |
| UPDRS Total Score | | 53.50 [40.75, 72.75] | 58.00 [53.25, 66.00] | | 56.00 [41.00, 67.50] | | 61.00 [57.00, 91.00] | | **0.038** |
| UPDRS_I | | 11.00 [7.00, 15.75] | 11.00 [10.00, 14.75] | | 10.00 [7.00, 15.00] | | 10.00 [7.00, 16.00] | | 0.919 |
| UPDRS_II | | 16.00 [12.25, 22.75] | 17.00 [16.00, 19.25] | | 19.00 [14.00, 21.50] | | 20.00 [16.50, 30.00] | | 0.083 |
| UPDRS_III | | 27.00 [18.00, 34.50] | 29.00 [23.00, 31.50] | | 24.00 [21.50, 37.50] | | 32.00 [25.50, 42.50] | | 0.109 |
| FOG-Q | | 12.50 [3.25, 19.00] | 12.00 [10.25, 14.25] | | 13.00 [10.00, 18.00] | | 16.00 [10.00, 20.00] | | 0.521 |
| H&Y Stage | | 3.00 [3.00, 3.75] | 3.50 [3.00, 4.00] | | 3.00 [3.00, 3.00] | | 3.00 [3.00, 4.00] | | 0.26 |
| **Cognitive function** | |  |  | |  | |  | |  |
| MoCA | | 17.00 [12.00, 20.75] | 18.00 [16.75, 20.00] | | 16.00 [11.00, 20.00] | | 13.00 [10.00, 18.00] | | **0.044** |
| Visuospatial/Executive | | 2.00 [1.00, 3.00] | 2.00 [0.75, 2.25] | | 2.00 [1.00, 2.00] | | 1.00 [0.50, 1.00] | | **<0.001** |
| Naming | | 3.00 [2.00, 3.00] | 3.00 [2.75, 3.00] | | 2.00 [2.00, 3.00] | | 3.00 [2.00, 3.00] | | 0.48 |
| Attention | | 4.00 [2.00, 5.00] | 4.50 [3.00, 5.00] | | 4.00 [2.50, 5.00] | | 4.00 [3.00, 5.00] | | 0.877 |
| Language | | 1.00 [1.00, 2.00] | 1.00 [0.75, 1.25] | | 1.00 [0.00, 1.00] | | 1.00 [0.00, 1.00] | | 0.071 |
| Abstraction | | 1.00 [1.00, 1.00] | 1.00 [0.75, 1.25] | | 1.00 [0.50, 2.00] | | 1.00 [0.00, 2.00] | | 0.71 |
| Memory | | 1.00 [1.00, 2.00] | 2.00 [0.75, 3.00] | | 1.00 [0.00, 2.00] | | 0.00 [0.00, 1.00] | | **0.009** |
| Orientation | | 5.00 [4.00, 6.00] | 4.00 [4.00, 6.00] | | 5.00 [2.00, 5.00] | | 4.00 [2.00, 5.00] | | **0.041** |
| MMSE | | 21.00 [17.00, 25.00] | 21.50 [18.75, 24.25] | | 18.00 [16.50, 23.00] | | 19.00 [15.00, 21.50] | | 0.157 |
| Orientation | | 7.00 [6.00, 9.00] | 7.00 [5.75, 8.00] | | 7.00 [4.50, 7.50] | | 7.00 [5.00, 7.50] | | 0.061 |
| Registration | | 3.00 [2.00, 3.00] | 3.00 [2.75, 3.00] | | 3.00 [2.00, 3.00] | | 3.00 [2.00, 3.00] | | 0.937 |
| Attention/Calculation | | 2.50 [1.00, 4.00] | 4.50 [3.50, 5.00] | | 2.00 [1.00, 4.00] | | 3.00 [1.00, 4.00] | | 0.398 |
| Recall | | 2.00 [1.00, 2.00] | 1.00 [1.00, 2.00] | | 2.00 [1.50, 3.00] | | 1.00 [1.00, 2.50] | | 0.327 |
| Language | | 7.00 [5.25, 8.00] | 5.00 [5.00, 6.00] | | 5.00 [4.00, 6.00] | | 6.00 [4.00, 6.50] | | **<0.001** |
| Visuospatial | | 0.00 [0.00, 1.00] | 0.50 [0.00, 1.00] | | 0.00 [0.00, 1.00] | | 0.00 [0.00, 0.00] | | **0.005** |
| **Non-motor symptoms** | |  |  | |  | |  | |  |
| NMSS-total score | | 51.00 [31.50, 80.00] | 61.50 [51.00, 89.25] | | 44.00 [36.50, 62.50] | | 53.00 [40.00, 91.00] | | 0.418 |
| Cardiovascular | | 0.00 [0.00, 1.00] | 0.00 [0.00, 1.25] | | 0.00 [0.00, 0.00] | | 0.00 [0.00, 0.00] | | 0.182 |
| Sleep | | 9.50 [5.25, 18.75] | 19.50 [14.25, 28.75] | | 9.00 [6.00, 12.00] | | 10.00 [2.50, 19.50] | | 0.215 |
| Mood | | 7.00 [0.00, 20.50] | 7.00 [0.00, 20.50] | | 7.00 [0.50, 13.50] | | 11.00 [1.50, 18.00] | | 0.849 |
| Perceptual | | 0.00 [0.00, 2.25] | 0.00 [0.00, 0.00] | | 0.00 [0.00, 0.00] | | 0.00 [0.00, 1.00] | | 0.753 |
| Attention | | 4.00 [1.00, 8.75] | 4.50 [3.00, 8.00] | | 6.00 [3.00, 15.50] | | 10.00 [4.50, 17.00] | | **0.012** |
| Gastrointestinal | | 6.00 [2.00, 13.00] | 4.00 [3.00, 7.50] | | 6.00 [4.00, 14.00] | | 11.00 [4.50, 14.00] | | 0.36 |
| Urinary | | 8.00 [0.00, 16.00] | 3.00 [2.00, 15.25] | | 8.00 [2.00, 13.00] | | 6.00 [1.00, 19.50] | | 0.999 |
| Sexual | | 0.00 [0.00, 0.00] | 0.00 [0.00, 0.00] | | 0.00 [0.00, 0.00] | | 0.00 [0.00, 0.00] | | 0.795 |
| Miscellaneous | | 1.00 [0.00, 4.00] | 0.50 [0.00, 6.50] | | 0.00 [0.00, 4.00] | | 1.00 [0.00, 9.50] | | 0.481 |
| HAH-D | | 12.90 (6.71) | 14.62 (9.46) | | 11.47 (5.45) | | 11.29 (5.69) | | 0.453 |
| HAM-A | | 11.00 [6.00, 16.00] | 13.50 [9.75, 18.50] | | 9.00 [6.50, 14.50] | | 9.00 [6.50, 16.50] | | 0.651 |

***** Continuous variables are shown as mean (standard deviation) or as median (interquartile range[P25–P75]). Categorical variables are shown as frequency (percent). Group comparison in continuous variables was performed using Student’s t test and Mann-Whitney U test. Chi-squared test and Fisher’s exact test were used for categorical variables. Significant difference was indicated in bold.

**Supplementary Table 2 Association between alcohol consumption and motor symptoms and cognitive functions**

|  | **Model 1** | | **Model 2** | | **Model 3** | | **Model 4** | |
| --- | --- | --- | --- | --- | --- | --- | --- | --- |
|  | **β (95%CI)** | **p** | **β (95%CI)** | **p** | **β (95%CI)** | **p** | **β (95%CI)** | **p** |
| **PSPRS** |  |  |  |  |  |  |  |  |
| Non-drinker | 0 (Ref) |  | 0 (Ref) |  | 0 (Ref) |  | 0 (Ref) |  |
| Light drinker | 1.114 (-7.015,9.243) | 0.787 | 2.608 (-6.02,11.237) | 0.551 | 3.942 (-4.901,12.785) | 0.380 | 2.22 (-6.247,10.688) | 0.605 |
| Moderate drinker | 4.035 (-1.599,9.67) | 0.159 | 5.617 (-0.984,12.218) | 0.095 | 7.582 (0.393,14.77) | **0.039** | 7.55 (0.707,14.393) | **0.031** |
| Heavy drinker | 6.453 (1.754,11.152) | **0.008** | 8.015 (2.528,13.503) | **0.005** | 10.696 (4.163,17.23) | **0.002** | 10.122 (3.889,16.355) | **0.002** |
| **UPDRS** |  |  |  |  |  |  |  |  |
| Non-drinker | 0 (Ref) |  | 0 (Ref) |  | 0 (Ref) |  | 0 (Ref) |  |
| Light drinker | 0.286 (-17.235,17.806) | 0.974 | 1.17 (-17.312,19.652) | 0.901 | 2.637 (-16.433,21.707) | 0.785 | 0.089 (-18.534,18.713) | 0.992 |
| Moderate drinker | 0.523 (-11.621,12.666) | 0.932 | 0.724 (-13.415,14.863) | 0.919 | 3.188 (-12.313,18.689) | 0.685 | 3.068 (-11.983,18.118) | 0.687 |
| Heavy drinker | 16.495 (6.368,26.623) | **0.002** | 16.763 (5.009,28.516) | **0.006** | 19.822 (5.733,33.911) | **0.006** | 18.443 (4.734,32.152) | **0.009** |
| **Moca** |  |  |  |  |  |  |  |  |
| Non-drinker | 0 (Ref) |  | 0 (Ref) |  | 0 (Ref) |  | 0 (Ref) |  |
| Light drinker | 0.411 (-3.086, 3.907) | 0.817 | -0.214 (-3.362, 2.933) | 0.893 | -0.732 (-3.947,2.483) | 0.653 | -0.386 (-3.597,2.824) | 0.812 |
| Moderate drinker | -1.429 (-3.76, 0.903) | 0.228 | -1.856 (-4.104, 0.392) | 0.105 | -2.336 (-4.793,0.122) | 0.062 | -2.307 (-4.746,0.132) | 0.064 |
| Heavy drinker | -3.266 (-5.335, -1.197) | **0.002** | -5.368 (-7.466, -3.271) | **< 0.001** | -6.124 (-8.595, -3.654) | **< 0.001** | -5.99 (-8.449, -3.53) | **< 0.001** |
| **MMSE** |  |  |  |  |  |  |  |  |
| Non-drinker | 0 (Ref) |  | 0 (Ref) |  | 0 (Ref) |  | 0 (Ref) |  |
| Light drinker | -0.304 (-3.996, 3.389) | 0.871 | -0.6 (-4.09, 2.89) | 0.734 | -0.895 (-4.481, 2.69) | 0.622 | -0.344 (-3.861, 3.173) | 0.847 |
| Moderate drinker | -1.738 (-4.2, 0.724) | 0.165 | -1.94 (-4.432, 0.553) | 0.126 | -2.039 (-4.78, 0.702) | 0.143 | -1.99 (-4.663, 0.682) | 0.143 |
| Heavy drinker | -3.135 (-5.32, -0.951) | **0.005** | -4.835 (-7.161, -2.509) | **< 0.001** | -5.285 (-8.041, -2.53) | **< 0.001** | -5.093 (-7.787, -2.398) | **< 0.001** |

Model 1: No adjustment; Model 2: Adjusted for demographic variables (age, gender, education level, and BMI); Model 3: Adjusted as in model 2, additionally adjusted for hypertension, diabetes mellitus, and smoking status; Model 4: Adjusted as in model 3, additionally adjusted for duration and phenotype.
